# Personal

**Published:** 1893-12

**Authors:** 


### Personal.

DR. GEORGE F. COTT announces his removal from 560 Michigan street to 43 W. Huron street. He will hereafter limit his practice to the treatment of diseases of the throat and nose. His office hours are from 10 A. M. to 1 P. M.

Dr. Henry J. Mulford announces his removal from 107 Delaware avenue to 56 Allen street. His telephone number is 599, and his hours are from 9 A. M. to 1 P. M.

---

Dr. A. B. Miller, of 326 Montgomery street, Syracuse, announces that his practice in the future will be limited entirely to the treatment of diseases of women, including abdominal surgery. He will, therefore, decline all general practice. Dr. Miller's skill in the lines he has adopted is well known.

